# “Is There Anything Else You Would Like to Add?”: The Ethics of (Not) Addressing Research Participants' Top Concerns in Public Health Emergency Health Research

**DOI:** 10.3389/fpubh.2022.796414

**Published:** 2022-01-26

**Authors:** Elysée Nouvet, Matthew Hunt, Lisa Schwartz

**Affiliations:** ^1^School of Health Studies, Western University, London, ON, Canada; ^2^School of Physical and Occupational Therapy, McGill University, Montreal, QC, Canada; ^3^Health Evidence, Impact and Methods, McMaster University, Hamilton, ON, Canada

**Keywords:** public health emergency research, ethics, advocacy, Ebola, West Africa

## Abstract

When conducting interviews or focus groups, researchers often end with a simple question; “Is there anything else you would like to add?” This article takes responses to this question provided by participants in a study of “West Africans' Perceptions of Ebola research” as its point of departure. A number of participants in that study accepted the invitation to add on to their interview at its end with details of suffering from the sequelae of Ebola in their communities, and criticisms of state social abandonment. Some explicitly asked the researcher to ensure the suffering of Ebola survivors would be recognized at the international level. These closing words exceeded the objectives of the study within which they emerged. This was a study focused on lived experiences and decision-making to participate in Ebola research during or after the 2013–16 West Africa Ebola outbreak. The study aimed to inform the ethical conduct of research in future public health emergencies. What to do, then, in the face of these participants' entreaties to the interviewer for action to address Ebola survivors' suffering and social abandonment? Can and should the public health emergency or qualitative researcher better anticipate such requests? Where participants' expressed concerns and hopes for the impact of a study exceed its intended scope and the researchers' original intentions, what is at stake ethically in how we respond to those entreaties as researchers? This paper offers reflections on these questions. In doing so, our intention is to open up a space for further consideration and debate on the ethics of how researchers respond to unanticipated requests made to them in the course of research projects, to leverage their power and privilege to advance local priorities.

## Introduction

The interview is almost over. *Margaret* (a pseudonym), a nurse in a hospital in Freetown, Sierra Leone, has been talking about her participation in a vaccine trial held during the 2013–16 Ebola outbreak. In doing so, she has had to recall a painful period: healthcare workers were particularly hard-hit by the disease, and her decision to receive an experimental vaccine was fraught and contentious. Now that the interview guide has been exhausted, the interviewer has one last thing to ask. Is there anything else that *Margaret* would like to say, about any of the topics they have touched on, or otherwise?

There is, but it does not relate to her own experience of research participation:

Well, because the most important thing [is] the survivors are here that Ebola virus hit, they are all over the country. Some of them, they are healthy. Some [have] problems with their eyes, their kidneys, they are having so many problems with their health. So we are asking some NGOs, some international organizations to come in their aid; so we are asking to help them.
Because for me, I'm not sick with Ebola. Those people out there who are suffering from this Ebola outbreak after they are getting treated, up to this time they are facing a lot of health challenges, so if there are any organization out there, let them come and help them. Some of them are having eye problems, kidney problems, some of them are suffering a lot, but if some of them listen to this interview, let them come to their aid, let them come help them. Some of them they lost their parents, they are not able to go to school, some of them don't have houses to sleep. But if anybody hear this interview, let them come to their aid and help them, please. Thank you.

As a matter of course, and like the majority of social scientists and interviewers, we usually end the interviews we conduct with some version of: “Is there anything else you would like to add?” This is both polite and practical: a way of checking back in with the participant that may lead to some final details or insights or points of emphasis. Often, and we say this based on 20+ years of conducting semi-structured interviews in our capacity as social scientists and qualitative researchers, the question simply stands for the close of the interview, and is frequently interpreted by the participant as an invitation to feel released from any further obligation to share more, to thank the interviewer for listening to their story, or to ask the interviewer some personal questions.

Sometimes, however, this question is taken up by study participants as an opportunity to communicate information that matters deeply but does not fit neatly with study objectives. *Margaret*'s call for action is an example. Her calm yet arresting plea was not at all unusual in the context of the “Perceptions and moral experiences of research participation during the 2014–16 Ebola outbreak” study (hereafter, referred to as the Perceptions Study) in which *Margaret* had agreed to speak with the first author. This article reflects on what such entreaties produce within the context of research with public health emergency affected populations, and considers the ethical obligations and options of researchers in response to requests for advocacy that extend beyond the intended scope, and arguably beyond the resources, of a research project. We begin by presenting an overview of the Perceptions Study. We connect our team's lack of preparedness for Ebola study participants' requests that our team leverage its international networks and privilege to help them advance their priorities to the limited co-design of our study. We outline how greater collaboration with Ebola survivors might have avoided our team being unprepared for this stakeholder group's requests.

Following consideration of shortcomings within our study's design, we then move into reflection on the possibilities and ethical implications of researchers responding in different ways to unanticipated requests by participants, namely that the researcher extend their intended activities to help the participants achieve their priorities.

## Background: This Study and its Limited Co-Design

The “Perceptions and Moral Experiences of Research Conducted during the West Africa Ebola outbreak” study was funded by an ELRA R2HC grant. Co-led by an international interdisciplinary research team, including four anthropologists, as well as ethicists and healthcare professionals, this qualitative study had as its goal to deepen understandings of challenges and strategies for the ethical conduct of research during public health emergencies. The authors of the current paper include the two co-principal investigators of the study and one co-investigator. The first author conducted many of the interviews undertaken as part of the Perceptions Study. The impetus for this paper comes from her experiences and reflections about what to do with responses to the “anything else to add?” query. In reflecting on issues of researcher roles and responsibilities, she engaged in discussion with the two co-authors, both of whom have extensive experience conducting qualitative research studies in the domain of public health emergencies. All three are based at universities in Canada.

The study was designed in response to needs identified by partners and others engaged in the work of using, or overseeing and regulating the use of, unproven treatments and prophylactics—the only treatments and prophylactics then available for the disease—during the 2014–6 West African outbreak. Guinea's *Comité National d'Éthique pour la Recherche en Santé* (National Health Research Ethics Board, or CNERS) was a partner on the project, and a local anthropologist, Sekou Kouyate^1^, played a key role in both data collection and analysis. The Ethics Research Board of Médecins Sans Frontières International (MSF or Doctors without Borders) also recognized the value of this study, as communicated in a letter of support for the project submitted with our application for funding. Our collectively developed plan was to use the study to develop the kinds of evidence that could be used to support decision-making by healthcare non-governmental organizations (NGOs), intergovernmental organizations, and research ethics boards. In our case, this meant trying to clarify and identify patterns within the experiences and perceptions of people who had engaged with research from different vantages, in different national and local contexts. We conducted 108 semi-structured interviews with a wide range of Ebola research stakeholders: participants in clinical trials and other Ebola studies conducted between 2014 and 2016 in Liberia, Guinea, and Sierra Leone; researchers; and, key research decision makers (e.g., government representatives, scientific committee members; survivors' association representatives). Primary findings with further details on methods have been reported elsewhere (1).

The study was developed in dialogue with some but not all relevant stakeholder groups. Ethically, we failed: to ensure those potentially most affected by our research had a say in the focus of our research. We did not leverage the full potential of the project to enact respect and recognition for those in our study structurally and socially positioned to be routinely excluded from the setting of research agendas, as widely recommended (2, 3). We did not form a community advisory board for the study, a known strategy for more inclusive and equitable research in public health emergency research contexts (4). While working closely with members of the National Health Research Ethics Committee of Guinea in our study design, we did not involve Ebola trial participants on our study team. Collaboration with Ebola research participants likely would have drawn attention to the importance of this stakeholder groups' concerns early on. Perhaps this would have shifted our study objectives.

Collaboration with Ebola survivors on the study's design might also have helped our team better prepare for interviewees requests that our team leverage its international networks and privilege to help this stakeholder group advance their priorities. Certainly, co-designing the study with input from Ebola research participants may have enabled us as a research team to anticipate and plan for requests for help and action beyond the conduct of research in public health emergencies. Such early conversation and collaboration with Ebola research participants might have also led to us explicitly ask those we interviewed to reflect on *our* study objectives, and the ethics of international researchers working to influence policy in one domain (in our case the domain of research ethics), when that domain is not the study participant's main concern. In retrospect, it is very clear to us that by not co-developing this study in dialogue with representatives from all the stakeholder groups we aimed to interview and serve through our study, we did lose an opportunity to explore additional questions relevant within a study on moral experiences of research. Moreover, in the absence of such co-design with Ebola trial/study participants, our team was caught somewhat off-guard by how much participants from this stakeholder group were adding at the end of their interviews, and by the consistent nature of the information added. So what can researchers do and what should researchers do in such instances? Do we have ethical obligations to respond with specific actions to entreaties for socio-political and material support when these have not been planned for at the time of a study's design? How do researchers' responsibilities depend on their position of power vis à vis those making such entreaties?

## “Yes, There Is Something I Would Like to Add”

Almost half of the 70 Ebola survivors with lived experiences of participation in Ebola research from whom we heard in this study ended their interview by describing the conditions faced by Ebola survivors or by others in Ebola-affected communities. A number of these descriptions involved, as did *Margaret*'s statement, decrying these conditions, and invoking or calling for international transformative action in response to local suffering.

Beyond the fact that all participants in the study had been touched by Ebola—as direct survivors of infection, as people whose families had suffered, or as members of heavily-hit communities and constituencies—and by Ebola research, the participants who made such statements harbored a range of social positions and experiences. A few were leaders and advocates from civil society organizations (Ebola survivors' associations). Some were healthcare professionals who may have been used to speaking for the communities they serve. Many were neither. This last group, the majority, included men and women with limited literacy working a range of jobs, most often in the informal sector. In the context of urban Guinea, Sierra Leone, and Liberia, as in many settings marked by social and economic class divisions, this last group has particularly limited resources—including limited social authority and opportunities based on connections—to shape understandings of the West Africa Ebola outbreak and its impacts on affected populations.

The statements made in the space created by the question “Is there anything you would like to add?” contained some differences as well as similarities. A few research participants called for specific measures. Participants already in positions of leadership or advocacy at the time of interview spoke, for instance, of wanting to see healthcare access guaranteed for all Ebola survivors throughout their lifetimes, or the introduction of capacity building programs to help survivors support themselves. Other participants, including healthcare professionals and limited literacy participants, spoke in fairly general terms, detailing what they framed as clear and pressing needs rather than on specific possible remedies. Many participants across all three categories simply described the situations that they had encountered: economic hardship following the loss of work while sick with Ebola, ongoing stigma in their families and communities as a result of their identity as an Ebola survivor, and their own or relatives' suffering from the physical sequelae of Ebola. These conditions were presented by participants as too hard to envision enduring long term, and for which solutions were needed. All categories of participants who spoke of suffering and needs at the end of their interviews described difficulties within their families, including economic or physical challenges not directly their own. The calls for aid were often simple and straightforward, but their anchoring in details and stories that the participants had faced or witnessed first-hand gave them additional power and made them especially compelling.

*Justin* (pseudonym) was interviewed as an Ebola survivor from Monrovia, Liberia. While acutely ill with Ebola, he had participated in a convalescent plasma study. Asked at the end of our interview if he had anything to add, his response was emphatic:

*Yes! I would like to say many things because, what I want to say is (…) at least let them try and cure something to start our life for a re-settlement. (…) Because during the crisis, our mattresses, our things, our clothes, (…) everything, during Ebola, when Ebola catch you, yeah, they will take all your things and burn it. So your home is empty. So I appeal to (…) our leader then, I told him (…) the international community help, the NGO, they need to help out with something to start our life, to make business, to sit down, (…) to make our life, to start our life. Because Ebola came and spoiled all (…) so now there is no foundation for us*.

*Justin* continued to explain that survivors had difficulty accessing any care, that many had died as a result, and that for all of the investment in Ebola research, there has been “nothing for us” survivors. He spoke quickly, jumping between and entwining what did happen and should not have with what did not happen but should have in his view.

*Bertrand* (pseudonym), a nurse and Ebola survivor from a mid-sized city in Guinea, shared stories of his experiences as a healthcare provider, as an Ebola patient, and as a participant in a study that provided free healthcare for survivors following the West Africa outbreak's official end. *Bertrand* finished the interview by bringing up his concerns about what would happen after the study's conclusion:

We have a child here today, if you tell him to walk over this way, he will walk over the other way. He is completely traumatized. His father and his mother are dead. So there are many. So there are children who cannot walk, who have no help from anyone, because everyone is dead. So there you have it: that is the problem that worries me a lot. And if there was to be help, it would really make me happy.

*Justin's and Bertrand'*s responses to the question “Is there anything you would like to add?” spelled out specific hopes that exceeded the objectives of our study and the impact our team had envisioned for the project. These responses spoke to experiences of marginalization, suffering, and need that were at once continuous with but also extended beyond the conditions and perceptions of Ebola research participation our study had set out to document. These statements revealed feelings of invisibility and limited power, but also unmet and pressing needs for material (economic and health) supports for Ebola-affected individuals, families, and communities. Ethically, what *should* be done with this information and entreaties? Is attentive, respectful listening a sufficient response?

As noted, our project was developed in a way that did not anticipate contributing to change in West Africa beyond practices related to the conduct of public health emergency research. And yet these statements urge us to question our relationship to participants and their calls for help. What relationships to us as researchers are participants outlining when they share with us such information or requests? What exactly are our responsibilities as researchers once we have been entrusted with these expressions of concern that are evidently central to our participants? And, are these responsibilities inherent or optional where researchers have means beyond those the participants in their study to draw attention to such concerns within global or local (and usually both historically and socially entrenched) structures of power?

## Possible Responses When Participants *Do* Have Something to Add

Statements such as those by *Margaret, Justin*, and *Bertrand* above could prompt us to reframe our understanding of the interviewee's agreement to an interview in important ways. They suggest that the decision to agree to an interview with our team might be intimately connected to a desire to speak about needs and hopes for action that lie beyond the goals of the project in which the interview is embedded. The positionality, perceived, and real relations of power between the interviewer and interviewee is important here. Interactions with researchers are shaped by structural and political factors (5). Two of the study's interviewers, including the first author, were white and Canadian. At the time of the interviews they held positions as assistant professors based at Canadian universities, and were responsible for overseeing data collection in Guinea, Sierra Leone, and Liberia. These white Canadian members of the team were assisted by three local social science trainees from the University of Sonfonia, in Guinea. The Canadian senior members of the team did conduct many of the interviews. When not directly conducting interviews, they remained close by: either sitting in on junior members' interviews, or conducting interviews at the same time as these local researchers in adjacent spaces. The very possibility of Canadians flying into West Africa to recruit participants and discuss with them their lived experiences of research communicated our team's connections to funding and networks extending beyond the localities participants inhabited. It is certainly possible, if not likely, that those agreeing to speak with our interview teams did so partially in the hopes that our team possessed, by virtue of connections to academic or other institutions or of their membership amongst a globally mobile “international community,” the power to act on the wrongs that participants identified: the power to fund capacity development programs for survivors, or at least to advocate effectively for such programs to be developed or funded.

As noted earlier, some of those interviewed in the study were not new to being invited to reflect or speak authoritatively on their own or others' experiences. As men and women with diverse positions of authority in families and communities, there is no reason to assume any of the study participants had not previously been asked to reflect on best practices or mediators of decision-making. Participation in an international study, however, represented for most a first opportunity to speak of their experiences in a public health emergency, and to individuals from outside their country positioned to circulate their statements internationally with potential impact on policy and practice. Before Ebola and the attention this brought to survivors such as *Margaret, Bertrand*, and *Justin*, such opportunities were structurally unlikely. Systems of political and social exclusion predated and characterized access to knowledge and power in Ebola-affected countries (6). Participants such as those cited in this paper are excluded from the spaces of knowledge production reserved for the elite within such systems. For some of the participants in the Perceptions Study, prevalent gender, age, and other power dynamics and hierarchies may also further limit opportunities to be involved in the production of knowledge which has the potential to inform policy and debate at the international level, but even at national and local levels. Speaking with an international researcher could understandably be interpreted by participants as a rare opportunity to have their concerns recorded and, perhaps, acted upon. There is more than one possible ethical response in this situation.

### Reiterating Study Goals

Where a participant indicates expectations of benefit or impact that exceed a study's objectives or what the interviewer regards as possible, the interviewer is generally understood to have an obligation to reiterate the goals of the study and its intended and expected scope of impact. Such scenarios remind us of the importance of clearly communicating a study's anticipated reach and limitations in terms of possible impact—something that is not clearly spelled out in many consent forms. Researchers have an obligation to be honest about the extent but also limits of their power, given the various strictures under which research is conducted, and when responding to questions participants may ask about the potential of the research to influence policy-makers and others. It is the researcher's responsibility to correct any misconceptions or misunderstandings they encounter on the participant's side, in terms of what their participation in research might achieve, when, for whom, on what bases. As interviewers, we did apply these normatively ethical responses to pleas for action we heard from participants at the end of interviews. We did so as part of respectful dialogue with our participants, and to avoid raising hopes and to avoid disappointments and disillusionments toward our team later. We did so also, because we did not feel it was within our capacity to achieve more than our study goals.

Under a strictly procedural understanding of what it means to engage in ethical research, requests from participants that exceed a project's scope do not ethically require a response beyond clarifying that such expectations fall outside the scope of the project. This is beyond consideration of legal obligations to report, which researchers do need to adhere to or negotiate. So, researchers who hear information about ongoing child abuse, for example, have legal obligations to report in many jurisdictions, including Canada. The above interpretation assumes that this sort of legal obligation is not present, and interviewees' statements are instead based on a misconception/misunderstanding of a study's scope of impact. If the problem is a misunderstanding, its remedy—information, communication—is relatively straightforward. Such rote normative research ethics “good practices” are simple enough to implement, if sometimes uncomfortable. But what if the source of participants' requests for further collaboration is not misunderstanding?

### Participants Recognizing Space to Advocate

A second possibility is a bit more complicated and harder to address. A person being interviewed for research can understand the study goals, the reasons they have been invited to interview, and yet still feel able, and maybe even morally obligated, to draw the researchers' attention to other questions and realities that fall outside the scope of their study. Participants can and did in our experience “get” why they were being interviewed (in our case, to document experiences and understandings of Ebola research participation—a set of perspectives that has been under-considered globally). That did not stop many of them from asking us to consider, in light of hardships presented as post-Ebola hardship, how our work might become more directly beneficial to their communities. These participants called our attention to realities that did not connect directly to our study, but that mattered deeply to them, and which we as researchers were positioned to share with audiences different than the ones they could reach. Entreaties by participants to widen the scope of our attention and help secure tangible assistance are not, in this second interpretation, based on misconceptions of our study goals at all. Indeed, that which participants choose to “add” at the end of interviews within this second perspective, could be seen as participants taking up what they regard as an opportunity to carve out greater benefit for their community, than a reflection of participants' misunderstandings. In this interpretation, what is at stake in responding or not responding to participants' requests that we as a research team extend our work to collaborate with them on their advocacy efforts?

## Power, advocacy, knowledge, responsibility

Public health researchers with positions in universities are empowered, through normative understandings of expertise contingent on educational attainment, track records of publication, and positions as paid “experts” in socially sanctioned institutions of knowledge production (primarily universities), to be heard when they speak. There are definite hierarchies of epistemic authority within academia and societies. While such hierarchies emerge through uneven access to opportunities for developing such expertise, and attribution of authority can vary greatly across and within disciplines, universities, and countries, academics *in general* are particularly well-positioned to secure attention and authoritativeness for their utterances. We may lament our limited readership, or get frustrated by the limited impact of our work on policy, practice, and thinking, but we are nevertheless socially anointed as experts to produce what is culturally sanctioned as “evidence.” In an era where “evidence-based” is an expected justification for change and action, academics' power to gather, and vet evidence, whether deserved or not, is meaningful. And yet, transforming the information participants such as *Justin* and others shared in the space of “anything to add” to produce the results he and other Ebola survivors seek is not a straightforward possibility here.

First, because the “things added” emerged at the tail end of interviews, and were not explored in depth with participants. Matters discussed at this point in the interview were not explored in depth. Considering these statements after data collection ended, we are concerned about the limits of what we know with respect to the preoccupations and recommendations for action outlined. We know that many Ebola-affected people and communities lack and need support, but we do not feel we know this in a way that would allow us to meaningfully inform, guide, or suggest action—at least not within the context of academic and applied academic scholarship.

Then, there is the question of how to frame integration of participants' entreatie. Merely transmitting or re-presenting participants' important claims and hopes by repeating them to academic audiences, as addenda to the more traditional research findings we may share, is one possibility. But it comes with risks: engaging in advocacy for social recognition and resources participants outlined wanting and needing could erode funders' or fellow academics' trust in our abilities to stay focused within our study goals and skill set as researchers.

Explicit advocacy within social science research arguably harbors risks. Within anthropology, 25 years ago, Scheper-Hughes (7) called for but also recognized the marginal status of social scientists unapologetically standing alongside research participants. Calling for action as a social scientist goes against a long history of equating the scientist's supposed detachment and neutrality with doing good research (7). Critics of Scheper-Hughes' push for the researcher/advocate have argued that taking clear stances on politically sensitive issues may do more harm than good: eroding the anthropologist's/researcher's trustworthiness in the eyes of decision-makers, given entrenched norms of equating sound research with neutrality (8). If calls for action are interpreted as biased, would this interpretation result in our overall analysis of West African Ebola research experiences being discounted as biased?

The study we set out to conduct to foreground lived experiences of Ebola research in West Africa was designed to produce the kinds of knowledge that would be “useable,” and recognizable as such, by researchers, policy-makers, and research ethics committees and regulators, in relation to research conducted during public health emergencies. Recruitment strategies, interview guides, and inter-disciplinary team-based analysis were developed to ensure that the conclusions we shared would be aligned with this objective.

Action for the sake of action also risks producing hollow gestures, whose value is purely symbolic and whose purpose is disconnected from the issues that participants sought to bring to our attention. Using our access to academic and other networks to pass on participants' words, without connecting participants to those networks, and without ensuring that what we say will be heard as meaningful, could become such a gesture, whose only real purpose/effect would be to act on and/or enact our power and status as public health emergency researchers. Doing so could be seen to reiterate an old Western hero framing that reproduces its own hegemony [e.g., (9), p. 430, response to (7)]. There is also—and this is a concern we had in writing the present piece—a risk that focusing on researchers' emotions (guilt, sense of responsibility), in a way that makes these objects of analysis themselves, draws attention away from the participants and their moral engagements.

If knowledge/engagement debates say something about why doing something feels risky, they also speak about why doing nothing feels wrong. In his call for an anthropological study of morals, [(10), p. 341–4] argues for the necessity of attending to interactions between researchers and research participants, as interactions between culturally/socially situated moral actors. In this logic, our own feelings of inadequacy and perplexity as researchers are telling and should be heeded and explored. They can serve as “a genuine research tool, which enables us to understand how our particular system of morals helps us to grasp or, sometimes, *prevents us from grasping*, moralities governing the life of the social groups we are observing.” [(10), p. 352, emphasis ours]. What kind of moralities are at play when participants speak to us, and what dimensions of our system of morals [and more broadly, of the academic and ethical apparatuses within which we are acting, cf. (11)] make it difficult for us to respond in kind?

We take seriously Scheper-Hughes' (7) argument that researchers have a responsibility to try to understand and engage with the struggles those participating in research face. We are familiar with concerns raised by others who have responded to her work, who argue that such engagement might undermine researchers' ability to perform a role as unbiased analyst. But it seems worth asking if the researcher's performance of neutrality is (always) ethical and appropriate, or merely conventional. As Fassin (12) and Stoczkowski (10) model, it would be best to unpack rather than frame in false dichotomies tensions between ethics and epistemology, engagement and knowledge. Doing so seems especially appropriate where these tensions pertain to researchers' attempts to understand, engage with, and become actors in, moral and political struggles that they are brought into contact with through their research.

To what extent does the context of participants' request to researchers matter to the researchers' obligations to respond? One of the things that the “Perceptions” study *did* set out to explore were participants' motivations for joining (or refusing to join) research studies (1). Many explained that they had joined studies in order to serve others, be it by donating plasma that might save the life of an Ebola patient, or by helping to test a vaccine that could potentially protect communities hit by future outbreaks. Participation in Ebola research was, as such, a moral act for many people (13, 14). Deciding to join or not to join a study enacted membership in a moral community [(15), p. 44]. As a reflection and affirmation of ties to other persons, it was an act discussed with, and sometimes advocated for amongst, other members of a moral community. Many participants explained how ethical concerns had informed the way they discussed research with their families and communities.

Often—very often—motivation to support clinical Ebola research as a participant was anchored in the participant's personal experience with, and firsthand knowledge of, Ebola. One man explained what motivated him to donate plasma, a gesture that he understood as potentially risky, but *necessary*, because:

Well, since I already knew the consequences of this disease, I knew how many people had died in front of me, so I wouldn't even wish this disease on an enemy. So I saw this. Since [plasma] was the first proposed treatment, that is why I had to participate.

(*Aboubacar*)

He also felt called on to act as an advocate, by “mobilizing” his family “to make sure that they would be vaccinated.”

This sense of responsibility borne from knowledge was characteristic of many participants in the “Perceptions” study. Often as a result of tragic events and great losses, but also of courageous and generous actions taken during the outbreak, many knew Ebola well. This deep knowledge stood in contrast to the Ebola denial that was widespread in many communities, especially during the early days of the outbreak, and to the slow international response to the outbreak. In other words: this deep knowledge stood in contrast to both ignorance about and/or indifference to the disease and its effects, or the people and communities it might (did) affect and the ways it might affect them. It became clear in our interviews as we asked about decision-making related to participation in clinical trials, that many felt that their hard-earned “expertise” had a moral weight as individuals who had lived Ebola infection and survived. Knowing Ebola implied a certain responsibility toward others. Agreeing to participate in a clinical trial related to Ebola, though sometimes terrifying and difficult, was a decision anchored in that sense of responsibility. As one participant explained: having survived, and having seen others die, meant that he could never “just stand there, with crossed arms” while others were still falling ill.

The ethical impetus to action implied by first-hand knowledge of suffering that emerged in the “Perceptions” interviews may provide a key to understanding why so many participants did respond in the way they did to the final interview question, “Is there anything you'd like to add?” Taking this question as an opportunity to speak of needs and identify means to mitigate further suffering beyond the scope of our project is consistent with a knowledge of suffering/action to try to mitigate suffering nexus found in many participants' explanations of why they had volunteered for Ebola research.

In this understanding, *Margaret, Justin, Bertrand*, and others can be understood to be engaging with our research project, to enact a moral sentiment that knowing about some suffering impels, ethically, trying to alleviate that suffering. In this emic perspective, for us as researchers to cast aside descriptions of need and entreaties to action as data “out of place,” and with no place in a presentation of findings, may feels particularly problematic.

## Methodological Exigencies—Ethical Conduct or Moral Folly?

There is an extent to which part of the problem falls within the requirements of the methods of academic research. The rigors of what is expected in the methodological process, data collection, and analysis help contain the research and give it some consistency within academic expectations, and help maintain focus and attention to an inquiry's established objectives, increasing the likelihood these will be achieved. This confers authoritativeness based on rigor, consistency and other desirable features. However, adherence to rigor can also erect borders affirming which interview content counts, and which does not. These borders may be acceptable and normative in some research, but they can also be regarded—and perhaps merit being rejected—as problematic.

Certainly, these feel artificial and morally distant from the person-to-person connection formed, if only temporarily, in the exchange that occurred in the context of the Perceptions study. This in turn raises questions about method and draws attention to the moral posture of the researcher, the obligations it create, and interpersonal responsibilities connected to unequal power between researchers and research participants.

In the face of this web of relationships, we might ask whether strict adherence to study objectives is appropriate in the context of this study, but even for qualitative research in general? Qualitative interviews are the best way to explore complex, often unexplored ideas, so it naturally opens unanticipated territory. The researcher can chose to “manage” moments that exceed a study's intended objectives, by politely acknowledging and then steering the interview “back on track” with further questions about the phenomenon of interest. But in the case being explored here, and given the exchange and relationships involved, it seems hard to call the comments made by *Margaret, Justin*, and *Bertrand* irrelevant. It is more the exigencies of academic limits that seem “irrelevant” in this moral context.

It feels wrong to do nothing with participants' entreaties to make survivors' suffering and needs heard, given in such entreaties participants such as *Margaret* are approaching us as fellow moral agents. They are inviting us into their moral community by sharing their knowledge. In the context of these participants affirming that for them knowledge and action are ethically inseparable, once the researcher as moral being holds the information, this information carries, at least for us, a weight of responsibility: an obligation to act rather than ignore. The appropriate thing to do is not to say—“this is out of the project requirements”—but instead to acknowledge that this is a finding that requires some form of response. At the very least, it seems fitting to include the information among the findings of the study either as an associated theme or as recommendations for further research or action.

We may not be able or willing to devote the time Ebola survivors' healthcare and social needs merit. As humans, however, we feel obligated to recognize these participants' moral sentiments by writing about them. The alternative seems ethically untenable. As Schepper-Hughes [(7), p. 418] argues, for anthropologists:

“Seeing, listening, touching, recording can be, if done with care and sensitivity, acts of solidarity. Not to look, not to touch, *not to record can be the hostile act, an act of indifference and turning away*.” [(7), p. 418].

Finding a place to share what participants in our study consider crucial to have us hear is ethically important beyond choosing concern over indifference. Thinking hard about these words spoken in the space of “Is there anything you would like to add?” feels crucial to defining our research endeavor as genuinely respectful of other ways of being in the world. Words spoken in that small space of the interview outline a moral logic: a shared understanding of the world as a place where when one knows about something that has caused or is causing suffering, one will do something toward its alleviation. To demonstrate respect for participants, it is necessary that we consider how we can document and disseminate such utterances, especially given we conducted these interviews to clarify what (un)ethical research means to those we interviewed. Ensuring participants' hopes for their engagements with research to result in change for their lives beyond the ethical conduct of research in emergencies gets recorded and shared in our study reports is something we can do.

The issue of power is key to thinking about what is produced in the space of “anything to add,” and about the ethics of how a research team respond to unanticipated entreaties for collaboration or advocacy. Ignoring matters of importance to participants strike us as particularly problematic in the context of a study designed with limited stakeholder input. Our team had already at the point of data collection failed to appropriately engage Ebola trial/study survivors in the co-design of the study. To only report on answers from Ebola survivors/study participants that mapped back to questions developed without input from this stakeholder group would further silence this group. Such silencing of under-heard groups lies at the heart of extractive research practices that are increasingly denounced in research with historically marginalized groups. As defined by Tilley, “[a]n extractive empiricist approach is, in part, one which assumes the right theory-guided questions are being asked, based on a prior assumption of sufficient knowledge about the field.” [(16), p. 38]. If we aim to distinguish ourselves from unethical extractive research, we need to practice being “guided at least partly by questions formulated by the participating community.” [(16), p. 38].

It is today widely recognized that being responsive to affected populations' priorities is key to good/ethical research in public health emergency research and indeed in all global health research, but arguably this is not in itself sufficient. It strikes us as equally important to be transparent and reflexive about that process of acknowledgment and its politics and ethics. Moving away from extractive research involves reflexive practice (16, 17). Practicing reflexivity is taken here to imply, “that the researcher should constantly take stock of their actions and their role in the research process and subject these to the same critical scrutiny as the rest of their “data.” [Mason in Guillemin and Guillan (18), p. 274]. As others before us have noted, the crux of ethical practice in qualitative research is not limited to ensuring international and general guidance are respected in protocols: ethical practice emerges in specific study contexts, through specific research events, in relationships, and in the decisions we make as researchers when faced with unanticipated situations or information in the course of conducting research (17–19). Unpacking the ethics of what to do in the face of unanticipated requests or findings that emerge as research unfolds means critically interrogating how and on what bases we feel compelled to respond in a particular way becomes part of that process. We do not just choose or not choose to report on particular concerns in relation to research objectives: inseparable from these scientific decisions is the power we have as researchers to make those decisions (17).

In ensuring participants' central concerns are reported, we do what many researchers working with marginalized populations do: we instrumentalize our authority and privileged positions in systems of knowledge, to act as agents of echolocation for research participants who do not have the same access to rendering their voices public (17, 20, 21). We acknowledge this remains an imperfect way forward. In this process, we reproduce the very systems of knowledge and exclusion that are part of Ebola survivors' limited access to being heard: colonial, historically entrenched, class, race-based, and linguistic systems of knowledge production that require so many in the world to rely on researchers as instruments through which they might increase their chances of being heard (17, 22).

A key take-away for our team from this experience relates to the crucial importance of engaging representatives from all—and not only some -stakeholder groups, when aiming for a co-created, context-relevant study. Such engagement seems especially important where qualitative research is undertaken on uneven geographies of knowledge production that make it difficult, if not impossible, for researchers to anticipate what will matter most to study participants based on published literature, or in conversation with colleagues who, while local, may also be disconnected from the realities of more marginalized stakeholders in a given research context.

## Conclusion

Knowledge can be generative of moral commitments, and shared knowledge of moral communities. When participants told us about the things that they needed to add, we *felt* obligated as researchers, as humans, and in reciprocity for their participation in the study, to listen. And yet we have struggled to clarify our obligations vis à vis responses to our question “Is there anything you would like to add?” Reflecting on our participants' responses to these questions has left us wondering about researcher obligations and relationships to participants' more generally. We could have ignored these responses: left them uncoded in our NVivo and parked them for oblivion. But to do so would reproduce the very exclusion of knowledges of people who had participated in Ebola research that our study had set out to address.

There may or may not be important differences in the ethics of taking a stand in one's research, dependent on whether one's work engages political conflicts that have already been defined as such. In the case of Scheper-Hughes, her consideration of what it meant to become or refuse a position of advocate occurred in a context of explicit political sides and agendas. We did not enter a world of clear political sides and agendas. Our research study was not at any point presented to potential participants as a project that would embark in advocacy efforts to transform existing conditions of economic, social, or healthcare need for survivors. But, not engaging with our participants' descriptions of daily hardships and need, feels wrong. It alerts us to the practical and ethical limits of defining our obligations to research participants based on a procedural understanding of researchers' obligations focused on ensuring voluntary and informed consent. Such an understanding protects us as researchers from any requests for relationships or benefits beyond those defined by the researcher and explained to potential participants in advance. Such a definition of our obligations as researchers normalizes extractive research: research that aims to pull out data based on the researcher's conceptualization of a problem, and which favors the flow of benefits from the knowledge it produces toward the researcher, rather than toward participants and their communities (11, 16). Working as anthropologists and qualitative researchers in global health, a field dedicated to foregrounding the uneven distribution of life and suffering, and global inequities in control over and access to resources, reproducing such extractive research does not feel ethical. As noted by Wright, “ethics also needs to take account of the political and structural factors that shape people's lives and their interactions with the research process” [(5), 516].

The spheres in which we have power and ability to act (academic scholarship, including engaged anthropology) do not readily recognize the kinds of knowledge imparted to us as ethically requiring a response. But knowledge is also circulated, assessed, and made meaningful within relationships and moral communities. As academics, we found ourselves struggling with how to meaningfully share and act on the knowledge we were given, and the obligations we were drawn into. It seems like even if we cannot change the social, economic, political conditions indicated by the participants, perhaps we can and should engage with these requests as a matter of respect and moral concern. Not necessarily as researcher to participant, but person to person. We hope our reflections here render available for further discussion and debate how academic norms (both evidentiary and ethical) shape the possibilities for developing the extended moral communities some participants might be aspiring to establish as they engage in research, and to considering how researchers might respond when participants do indeed have “something else to add.”

## Data Availability Statement

The data analyzed in this study is subject to the following licenses/restrictions: We did not obtain permission to render datasets publicly available from the Ethics Review Boards that approved the study. The corresponding author can be contacted to discuss access to data options. Requests to access these datasets should be directed to enouvet@uwo.ca.

## Ethics Statement

The studies involving human participants were reviewed and approved by Hamilton Integrated Research Ethics Board (Canada); Comité National d'Ethique en Recherche de la Santé (Guinea); Sierra Leone Ethics and Scientific Review Committee; University of Liberia Ethics Review Board. The patients/participants provided their written informed consent to participate in this study.

## Author Contributions

EN prepared the initial draft of this article. MH and LS provided critical feedback to refine its arguments. All authors contributed to revisions and approved the final manuscript.

## Funding

The research upon which this article is based was funded by Elrha's Research for Health in Humanitarian Crises (R2HC) programme (grant #21174). Elrha's R2HC programme aims to improve health outcomes by strengthening the evidence base for public health interventions in humanitarian crises. R2HC is funded by the UK Foreign, Commonwealth and Development Office (FCDO), Wellcome, and the Department of Health and Social Care (DHSC) through the National Institute for Health Research (NIHR).

## Conflict of Interest

The authors declare that the research was conducted in the absence of any commercial or financial relationships that could be construed as a potential conflict of interest.

## Publisher's Note

All claims expressed in this article are solely those of the authors and do not necessarily represent those of their affiliated organizations, or those of the publisher, the editors and the reviewers. Any product that may be evaluated in this article, or claim that may be made by its manufacturer, is not guaranteed or endorsed by the publisher.

## Footnotes

^1^Sekou Kouyate died from sepsis complications on December 16, 2020.
